# Report on the Diagnosis of Epidemic Cholera, Made to the Buffalo Medical Association

**Published:** 1849-08

**Authors:** Austin Flint, William Treat, John S. Trowbridge


BUFFALO MEDICAL JOURNAL

AND

MONTHLY REVIEW.

VOL. 5. AUGUST, 1849. NO. 3.

### ORIGINAL COMMUNICATIONS.

---

ART. 1.—*Report on the Diagnosis of Epidemic Cholera, made to the Buffalo Medical Association, and communicated for the Buffalo Medical Journal, in accordance with a resolution of the Association.*

The Buffalo Medical Association met on the evening of the 10th ult., to discuss the diagnostic criteria of Epidemic Cholera, and to settle upon an uniform system of reporting cases to the Board of Health. At that meeting a Committee was appointed to prepare a report, and present it at an adjourned meeting on the evening of the 12th ult. At the latter meeting the following report was adopted, and its publication in the columns of this Journal requested. It is due to the Committee, to direct the attention of the reader to the fact of its being prepared in an interval of forty-eight hours between the two meetings of the Association, and amidst the increased duties and anxieties incident to the prevalence of an epidemic disease. This should suffice as an apology for a want of elaborateness in the execution of the duty assigned to them, which, under other circumstances, would be less excusable.—EDITOR.

*Report.* The committee appointed at the meeting of the Association on the evening of the 10th inst., "to draft a report expressive of the sense of the Association relative to the objects of the meeting," having devoted to the duty assigned to them as much attention as their other engagements, during the 48 hours since the meeting, would admit of, beg leave, respectfully, to submit the following report:

The objects of the meeting of the Association on the evening of the 10th inst., as understood by your committee, were to discuss the circumstances appertaining to epidemic cholera, which are involved in the early discrimination of the disease, and to unite upon distinguishing traits, so as to secure uniformity of system among the practitioners of the city, in reporting cases to the Board of Health, in conformity with the requisitions of the municipal government.

The chairman of this committee, on whom devolves the labor of drafting the report, was unable to be present at that meeting, and, hence, he labors under the embarrassment of not having the advantage of an interchange of views by different members of the Association on that occasion. The very short period allowed for the preparation of the report, moreover, precludes satisfactory consultation with his associates of the committee, and other members of the association, in order to obtain information of the opinions generally entertained respecting the objects of that meeting. What may be submitted, therefore, will perhaps require renewed discussion, and more or less alteration, before becoming " expressive of the sense of the Association."

The objects of the meeting relate, primarily and chiefly, to the *diagnosis* of epidemic cholera. For the most part, they are embraced in the answer to the question, what symptoms are essential to the diagnosis of the disease in its incipiency or early stage? Upon the phenomena which belong to the descriptive history of epidemic cholera, the committee need not amplify; they are sufficiently characteristic and familiar. The diagnosis when most, or several of these phenomena are present, offers no difficulty. The consideration of cases in which the characters of the disease are well marked, does not enter the objects proposed by these meetings of the Association. The question is, where are the lines of demarkation separating epidemic cholera from other affections with which it is connected by the relation of antecedence and sequence, or to which it bears resemblances which may occasion error of diagnosis. The committee will limit themselves to the subject of diagnosis in this aspect, and endeavor to consider the subject wholly in a practical point of view. Here the diagnosis is not always easy, and may occasion considerable embarrassment. As practitioners, we know by experience that this is the case, and it is the conviction of this fact which has led to the calling together of the Association for the objects proposed.

Collective observations have ascertained, that an attack of epidemic cholera, in the vast majority of cases, is ushered in by ordinary diarrhœa, of a duration varying from a few hours to several days. It is not to be forgotten that this is not an invariable law of the disease. As a general rule, it holds good, but there are exceptions to the rule. Some practitioners regard this diarrhœa as constituting the first stage of the disease. We do not concur in this opinion. We conceive that, scientifically, this disease cannot be said to be present until some of its distinguishing traits are appreciable. At the same time, the practical importance of regarding the connection of the disease with the previous diarrhœa must be fully admitted, and for popular effect, to ensure greater prudence, there is an utility of considering them as essentially connected. But the practical result of considering cholera, and the antecedent diarrhœa, as different stages of one disease, would be, that we should be bound to consider and report all cases of diarrhœa, during the prevalence of the epidemic, as cases of cholera. Now we know that, during the prevalence of the epidemic, we daily meet with a host of cases of diarrhœa, which are not only relieved by simple remedies, but which in many instances cease spontaneously. Some end in cholera, and some do not, nor have we any criteria for determining positively, beforehand, what the termination would be, even without medical treatment. Then we arrive at the question in those cases in which the diarrhœa does merge into cholera, at what juncture does the latter disease begin—where is the dividing line between the premonitory symptoms and the disease itself?

Again, epidemic cholera is allied in resemblance, if not in reality, with sporadic cholera, or cholera morbus. Cases of the latter co-exist with the former, and are more numerous than at other periods. It is altogether probable that cases of sporadic cholera may eventuate in the epidemic disease. Now, how are we to discriminate with precision between the two? This is another point occasioning doubt in same instances.

These difficulties are somewhat enhanced by the fact, that the diagnosis, even in strikingly marked cases of epidemic cholera, is not based on any symptom which is invariably present, but on the aggregate of symptoms. Vomiting is not infrequently absent; diarrhœa, in a small ratio of cases, is not present. Indeed, cases are related in which choleraic symptoms, exclusive of those referrible to the alimentary canal, have been present without either vomiting or purging, [see New Orleans Medical and Surgical Journal for July 1846, art. by Dr. Delerey.] A copious and peculiar perspiration has sometimes appeared to take the place of these, generally, most characteristic of the phenomena belonging to the disease. Cramps are occasionally not observed, and, not unfrequently they are not prominent, nor are they peculiar to epidemic cholera. So, of morbid coldness of the surface, cyanosis, etc.

Not to extend these remarks, and waiving, on this occasion, discussion of the pathological questions connected with the facts just adverted to, your Committee would offer as the results of the reflection and observations bestowed on the subject before them, the following general principles as applicable to the class of cases referred to, in which, from the circumstances mentioned, the diagnosis is liable to involve doubt and difference of opinion.

1. It should be regarded as essential, in order that a case be considered one of epidemic cholera, either that one or more of the distinguishing features of the disease be present, or, that there be aggregated several of the symptoms which are more or less peculiar to the disease. In other words, to vary the mode of expression, it is not sufficient for diagnosis that the phenomena present are those which are generally prodromic of the disease, or bear a similitude to those distinctive of it; but there should be some positive, present evidences of the epidemic exhibited. It is very often the case that we are called upon to prescribe for ailments which, neglected, or not relieved, would merge, or eventuate in cholera; these are cases in which the disease is averted, not cured. We have no certain data, in such instances, for the conclusion that cholera would have ensued. It is a matter of opinion, or supposition, which, however rational or probable, is widely different from an established matter of fact. If this rule be not fully appreciated or observed, it will follow that many cases will be reported as cases of cholera incorrectly, and statistics of the epidemic more or less vitiated.

2. Of the phenomena incident to epidemic cholera in its early stage, the most constant, distinctive and reliable, are those which appertain to the intestinal evacuations. These are variable, but, at the same time, present peculiarities rarely found in connection with other diseases, and which are sufficiently appreciable. The characteristic points are, in the first place, negative, consisting in the absence (or nearly so) of the natural fecal odor, and of those appearance generally attributed to the presence of bile, but probably due to the cessation of all the normal intestinal secretions. The evacuations are devoid of feculent appearance and smell. The positive appearances are not uniform. The most common are serous, or rice water, usually commingled with flocculi, or small specs of fibrine; milky; turbid, in some cases, closely resembling gruel. These are, of course, familiar.

Now it is undoubtedly true that epidemic cholera may exist and prove fatal without presenting any of these symptoms; such cases are rare. The fact of their occurrence does not, in the least, invalidate the significancy of the symptoms, when present, as diagnostic criteria. Their importance, in this point of view, depends on the fact that they are peculiar to the disease under consideration, i. e. they are not observed in connection with other diseases.

Any case, during the prevalence of the epidemic, in which the alvine discharges are found to have lost all feculent qualities, and to exhibit some of the appearances just mentioned, in the opinion of your committee, is fairly entitled to be considered and reported as a case of cholera. The concurrence of vomiting, cramps, prostration, etc., are not essential to the diagnosis. There are generally secondary, and probably dependent phenomena. The gravity of the case is unimportant, for this usually results from the continuance of the disease, and is not essential to it, especially in the incipient stage. Judicious remedies, doubtless, promptly and early applied, will, in a large proportion of instances, speedily arrest the progress of the disease. This should not invalidate the diagnosis; and it is but justice to our art that this point be fully understood. The disease assuredly is not less cholera because it is curable early in its career, albeit medicine becomes almost powerless in a subsequent stage.

The discharges in cholera occasion no smarting or burning pain, in passing the rectum. This is to be recollected as having some value in diagnosis, where we have to obtain information from others respecting the evacuations. The importance of caution in drawing deductions from the description of appearances by friends or patients, is too obvious to require comment.

3. Next to the intestinal discharges, in diagnostic importance in cases of any doubt, are the gastric symptoms, as denoted by the evacuations by vomiting. Here the appearances are far less distinctive, because, in the first place, they are, to a greater extent, influenced by what has been ingested; and, in the second place, they are by no means so peculiar to the disease, i. e., they are observed under other morbid conditions. Not to dwell on this point, it may be mentioned, that the circumstances which appear to characterize, in some measure, the vomiting of cholera are, viz: (*a*) the whey, rice, or starch-like appearance of the fluid ejected; (*b*) the absence of the usual odor of substances vomited; (*c*) the forcible nature of the act of vomiting, and its occurring with but little nausea, or notable spasmodic pains. These circumstances denote an effusion into the gastric cavity; the absence of inflammation, or intense irritation; and the excitation of vomiting by an unirritating fluid, simply in consequence of mechanical distension of the organ. In view of the comparatively less distinguishing character of the symptoms appertaining to the evacuations by vomiting, your committee are of opinion, that in cases in which vomiting is present, without diarrhœa, a diagnosis should seldom, if ever, be based on the gastric symptoms alone; but the concurrence of other and secondary symptoms should be considered requisite — such as cramps, cyanosis, cadaverous feeling of skin, notably feeble or absent pulse, suppression of urine, etc. The instances in which cholera exists without purging, however, are so infrequent, that occasions for doubt respecting the value to be attached to vomiting, disconnected from the character of the intestinal evacuations, practically, will very seldom occur.

4. The train of phenomena belonging to the disease, exclusive of those just considered, are, for the most part, successive to the latter in the order of their occurrence, and are, probably, in a great measure, dependent thereupon, being due, as is generally supposed, chiefly to the serous effusion. They confirm, of course, the diagnosis, and when many of them occur, and in a marked degree, render discrimination as easy as it is certain. But it is well established, as already stated, that this train of phenomena may occur without any purging, and perhaps even without vomiting. The practitioner is seldom exposed to the hazard of mistaking such cases, inasmuch as the ratio of their occurrence is infinitely small. Still, it is to be recollected that authentic cases have been reported in which the group of symptoms characterizing the stage of collapse (as it is generally styled,) has been present, without any dejections having occurred. Dissections in fatal cases of this description, have revealed the retention of serous effusion within the intestinal tube. Such cases are like cases of concealed hemorrhage of the uterus in the puerperal state.

To justify a diagnosis in the instances just referred to, your committee are of opinion that there should be present an aggregate of the most marked of the secondary symptoms, to wit: coldness of the surface, cyanosis, spasms, suppression of urine, etc,

As regards the prodromic, or premonitory symptoms of epidemic cholera, it might, a priori, or from analogy, be suspected that some diagnostic elements might be embraced among them. Analysis and comparison, however, disclose nothing here which can be relied upon as distinctive. These symptoms are essentially similar to those which may precede an attack of sporadic cholera, or simple diarrhœa, and their value as criteria are consequently lost.

One point occurs to your committee to suggest to the Association in this connection, as a point which may perhaps have some little importance in a diagnostic view. It is suggested as a matter of inquiry, the committee not having time and opportunity to consult authorities on the subject. On referring to personal experience, the impression is entertained that sporadic cholera is seldom preceded by diarrhœa, but that the vomiting and purging are equally abrupt in their occurrence. Epidemic cholera, on the other hand, is (as is well known,) almost invariably preceded by diarrhœa. There are other obvious points involved in the differential diagnosis between sporadic and epidemic cholera, upon which it is not necessary to dwell on this occasion. The point to which the attention of the Association is invited, we do not remember to have seen alluded to by writers on this subject.

The importance of determining, in so far as may be, well defined diagnostic limits of the epidemic under consideration, is doubtless as fully appreciated by every member of this Association, as it is by your committee. Correctness of judgment in diagnosis is essential to render the experience of the practitioner of value to himself, or of service to the profession. Without accuracy in this respect, statistics with reference to the prevalence of the disease, the ratio of its mortality, and the efficacy of treatment, are worthy of little or no confidence. It is by wantonly disregarding all limitations of diagnosis that arithmetic has been invoked to sustain the waning popularity of the *meanest* of all systems of imposition upon public credulity. It is needless to say that we allude to Homœopathy. It is in keeping with a system of deception, which, under the guise of fashionable dogmas, avails itself, in so far as its partizans are capable, of the resources of legitimate medicine, to attempt to defraud the judgment of the credulous, by resorting to false statistics.

In conclusion, your committee submit the following resolutions, as embodying, in brief, the rules of diagnosis imperfectly set forth in the foregoing remarks:—

*Resolved,* That, in the opinion of this Association, the occurence of intestinal discharges devoid of feculent appearance and odor, and resembling rice water, whey, milk, or milk and water, or gruel, containing opaque floculi, or specs, is sufficient to authorise the conclusion that the disease is epidemic cholera, during the prevalence of the disease, without reference to the associated symptoms, or the facility with which the farther progres of disease is arrested.

*Resolved,* That the occurrence of vomiting, whatever may be the appearance of the matter vomited, is not sufficient to establish the existence of epidemic cholera, unless characteristic intestinal discharges are also present; or unless the ensemble of symptoms belonging to the average of well marked cases of cholera, exclusive of the symptoms appertaining to the alimentary canal, are also present.

*Resolved,* That in the absence of vomiting or purging, or an inability to ascertain, definitely, the qualities of the matter discharged from the stomach and intestines, or in the event of these matters not being characteristic, the group of symptoms belonging to the average of well marked cases of cholera in the second stage must be present, to authorise a positive diagnosis.

The committee will add another resolution, which has been suggested by a respected senior member of the Association, not a member of the committee, (Dr. Josiah Trowbridge). The objeet of the resolution is to secure the record of sufficient data in the reports of cases, for the Board of Health, and others, to draw therefrom their own conclusions.

*Resolved,* That the Board of Health be recommended by this Association to request practitioners to enumerate in their daily report of cases, briefly, the symptoms present in each case, upon which their diagnosis is based.

All which is respectfully submitted,

AUSTIN FLINT,
WILLIAM TREAT.
JOHN S. TROWBRIDGE.

